# Supplementary material for: Identification of photoperiod-induced specific miRNAs in the adrenal glands of Sunite sheep (Ovis aries)
Source: Front Vet Sci. 2022 Jul 22;9:888207. doi: 10.3389/fvets.2022.888207 (PMC9354845; doi:10.3389/fvets.2022.888207)
Supplement: Supplementary file 1 [file Data_Sheet_1.ZIP › Supplementary file/miRDeep2 file.zip!/miRDeep2 file/Know_Structure/SPLP42A2/oar-mir-134.pdf]

miRBase precursor : oar-mir-134
Total read count : 142
oar-miR-134-5p read count : 129
oar-miR-134-3p read count : 13
remaining reads : 0

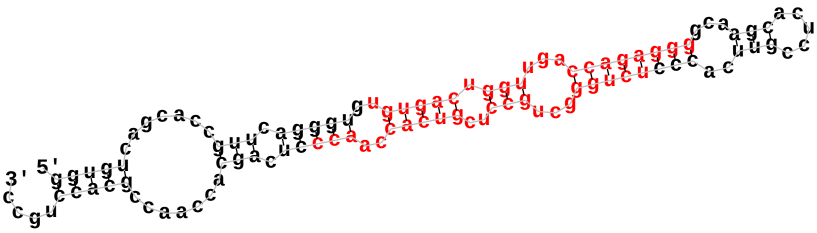

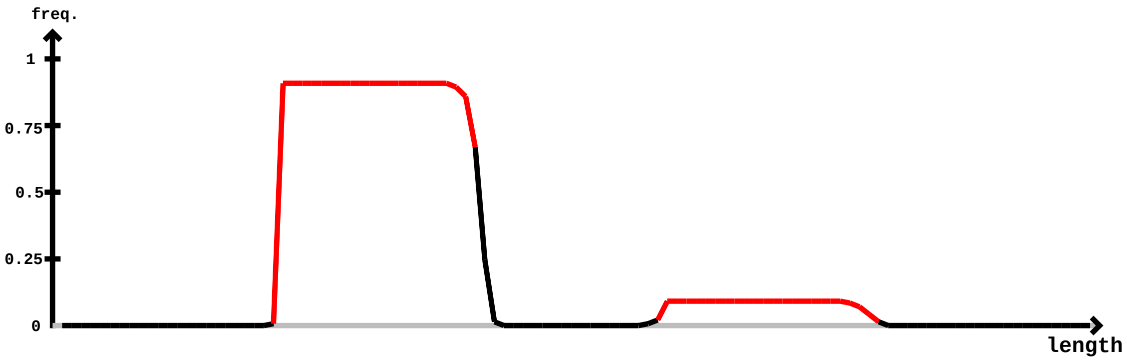

oar-miR-134-3p

oar-miR-134-5p

```
5'- ggugucagcaccguucagggugugugacugguugaccagagggcaagcacuccguucacccucugggcugccucgucaccaacccucagcaccaaccgcaccugcc   -3'    exp
    (((((.......(((.(((((..(((((.(((...((((((((...(((.....)))..))))))))...)))..)))))..))))).))).......))))....   reads       mm          sample
    ...................gugugacugguugaccagagg...................................................................   1           0           seq
    ......................ugugacugguugaccaga...................................................................   2           0           seq
    ......................ugugacugguugaccagag..................................................................   5           0           seq
    ......................ugugacugguugaccagagg.................................................................   26          0           seq
    ......................ugugacugguugaccagaggg................................................................   60          0           seq
    ......................ugugacugguugaccagagggg...............................................................   33          0           seq
    ......................ugugacugguugaccagaggggc..............................................................   2           0           seq
    ............................................................ccucugggcugccucgucacc..........................   1           0           seq
    .............................................................cucugggcugccucgucacca.........................   1           0           seq
    .............................................................cucugggcugccucgucaccaa........................   1           0           seq
    ..............................................................ucugggcugccucgucacca.........................   1           0           seq
    ..............................................................ucugggcugccucgucaccaa........................   3           0           seq
    ..............................................................ucugggcugccucgucaccaac.......................   4           0           seq
    ..............................................................ucugggcugccucgucaccaacc......................   2           0           seq
```
